# PARP1 Inhibitor and Trabectedin Combination Does Not Increase Tumor Mutational Burden in Advanced Sarcomas—A Preclinical and Translational Study

**DOI:** 10.3390/cancers13246295

**Published:** 2021-12-15

**Authors:** Ymera Pignochino, Giovanni Crisafulli, Giorgia Giordano, Alessandra Merlini, Enrico Berrino, Maria Laura Centomo, Giulia Chiabotto, Silvia Brusco, Marco Basiricò, Elena Maldi, Alberto Pisacane, Valeria Leuci, Dario Sangiolo, Lorenzo D’Ambrosio, Massimo Aglietta, Bernd Kasper, Alberto Bardelli, Giovanni Grignani

**Affiliations:** 1 Department of Clinical and Biological Sciences, University of Torino, 10100 Torino, Italy; ymera.pignochino@unito.it; 2 Candiolo Cancer Institute, FPO-IRCCS, 10060 Candiolo, Italy; giovanni.crisafulli@unito.it (G.C.); giorgia.giordano@unito.it (G.G.); enrico.berrino@unito.it (E.B.); marialaura.centomo@unito.it (M.L.C.); silvia.brusco@unito.it (S.B.); marco.basirico@ircc.it (M.B.); elena.maldi@ircc.it (E.M.); alberto.pisacane@ircc.it (A.P.); valeria.leuci@ircc.it (V.L.); dario.sangiolo@unito.it (D.S.); LDambrosio@asl.at.it (L.D.); massimo.aglietta@ircc.it (M.A.); alberto.bardelli@unito.it (A.B.); giovanni.grignani@ircc.it (G.G.); 3 Department of Oncology, University of Torino, 10100 Torino, Italy; 4 Department of Medical Sciences, University of Torino, 10100 Torino, Italy; giulia.chiabotto@unito.it; 5 Cardinal Massaia Hospital, 14100 Asti, Italy; 6 Sarcoma Unit, Mannheim University Medical Center, University of Heidelberg, 68167 Mannheim, Germany; bernd.kasper@medma.uni-heidelberg.de

**Keywords:** advanced sarcomas, tumor mutational burden, DNA damage response and repair genes, trabectedin, olaparib, mutational signatures

## Abstract

**Simple Summary:**

Immunotherapy has revolutionized cancer treatment, but not for all tumor types. Indeed, sarcomas are considered “immune-cold” tumors, which are relatively unresponsive to immunotherapy. One strategy to potentiate immunotherapy efficacy is to increase tumor immunogenicity, for instance by boosting the number of candidate targets (neoantigens) to be recognized by the immune system. Tumor mutational burden indicates the number of somatic mutations identified in the tumor and normalized per megabase. Tumor mutational burden is considered as an acceptable, measurable surrogate of tumor neoantigens. Here, we explored whether the combination of two DNA-damaging agents, trabectedin and olaparib, could increase tumor mutational burden in sarcomas, to prime subsequent immunotherapy. We found no variation in tumor mutational burden after trabectedin + olaparib in preclinical and clinical samples. Therefore, other aspects should be considered to increase sarcoma immunogenicity, by exploiting different pathways such as the potential modulation of the tumor microenvironment induced by trabectedin + olaparib.

**Abstract:**

Drug-induced tumor mutational burden (TMB) may contribute to unleashing the immune response in relatively “immune-cold” tumors, such as sarcomas. We previously showed that PARP1 inhibition perpetuates the DNA damage induced by the chemotherapeutic agent trabectedin in both preclinical models and sarcoma patients. In the present work, we explored acquired genetic changes in DNA repair genes, mutational signatures, and TMB in a translational platform composed of cell lines, xenografts, and tumor samples from patients treated with trabectedin and olaparib combination, compared to cells treated with temozolomide, an alkylating agent that induces hypermutation. Whole-exome and targeted panel sequencing data analyses revealed that three cycles of trabectedin and olaparib combination neither affected the mutational profiles, DNA repair gene status, or copy number alterations, nor increased TMB both in homologous recombinant-defective and proficient cells or in xenografts. Moreover, TMB was not increased in tumor specimens derived from trabectedin- and olaparib-treated patients (5–6 cycles) when compared to pre-treatment biopsies. Conversely, repeated treatments with temozolomide induced a massive TMB increase in the SJSA-1 osteosarcoma model. In conclusion, a trabectedin and olaparib combination did not show mutagenic effects and is unlikely to prime subsequent immune-therapeutic interventions based on TMB increase. On the other hand, these findings are reassuring in the increasing warning of treatment-induced hematologic malignancies correlated to PARP1 inhibitor use.

## 1. Introduction

Immune-checkpoint inhibitors (ICI) have demonstrated a striking degree of efficacy in several advanced tumors, prompting a dedicated effort to identify predictive factors of response [1]. As of today, immune-infiltrate, T-cell receptor clonality, specific gene expression signatures have been associated with clinical response [2,3,4]. Moreover, tumor mutational burden (TMB), defined as the number of somatic mutations normalized per megabase, correlates with ICI activity [5,6], supposedly because it leads to the production of a larger set of tumor neoantigens, increasing the probability of triggering a specific T-cell clonal response. In this perspective, drugs generating DNA damage might induce mutations resulting in the production of neoantigens [7,8,9], despite the fact that these neoantigens may be related to subclones of the tumor (branched) and not to clonal mutations (truncal) that are more evenly expressed on all tumor cells and are correlated with a better response to ICI [10]. In the context of pharmacologically induced DNA damage, we focused on PARP1 inhibitors (PARP1i). Regardless of their use, PARP1i were developed to hit tumor cells bearing genetic (germinal or somatic) DNA damage response/repair defects or chemotherapy-induced ones [11]. Indeed, impairment of base excision, nucleotide excision and homologous repair mechanisms promotes the activation of the error-prone non-homologous end-joining (NHEJ) DNA repair machinery, eventually favoring the accumulation of mutations in tumor cells [12]. Inactivating mutations of key components in DNA damage response and repair (DDRR) pathways can lead to high TMB in tumor cells [13,14]. For instance, in ovarian cancers bearing BRCA1/2 defects, TMB is higher if compared to BRCA1/2 wild-type tumors [15]. Moreover, PARP1i stimulates immunomodulatory pathways in cancer cells and may reshape the tumor microenvironment [16]. These findings have raised interest in the potential of combining PARP1i with ICI; several trials are ongoing to explore this hypothesis [17]. The dark side of a TMB increase is the potential induction of secondary tumors, especially hematological ones. Indeed, the wider use of PARP1i brought about a small, but significantly increased, risk of developing myelodysplastic syndromes or leukemia, as evidenced by randomized clinical trials and a recent meta-analysis [18,19].

In search of clinically feasible PARP1i partners, we and others showed that the antitumor activity of trabectedin was increased by PARP1 inhibition in sarcoma preclinical models [20,21,22]. Thereafter, we ran a phase Ib trial on the combination of trabectedin and olaparib in patients affected by advanced bone and soft tissue sarcomas, proving feasibility and showing preliminary signs of activity [23]. Since bone and soft tissue sarcomas are characterized by an averagely low TMB and fail to display significant sensitivity to ICI [24], we investigated whether this combination might increase TMB, so as to represent a priming intervention for further integration with immunotherapy, thanks to the appearance of tumor-specific neoantigens capable of activating T-cell responses. In this work, we evaluated DDRR mutational status, mutational signatures, and TMB after treatment with trabectedin and olaparib in a preclinical and translational platform of bone and soft tissue sarcoma cell lines, xenografts models, BRCA-proficient and defective cells and in temozolomide-treated cells as a positive control. Moreover, we took advantage of tumor samples derived from patients enrolled in the phase Ib TOMAS trial testing the trabectedin and olaparib combination in advanced sarcomas to further confirm our preclinical findings. We showed that the trabectedin and olaparib combination did not affect mutational signatures, DDRR gene status, gene copy number, clonality, and TMB. These findings indicate that trabectedin and olaparib combination did not show mutagenic effects and is unlikely to prime subsequent immune-therapeutic interventions based on TMB increase, while potentially reassuring the warning of myelodysplastic risk.

## 2. Materials and Methods

### 2.1. Cell Cultures, BRCA1 Stable Silencing and Drug Treatment

**Figure 1:**
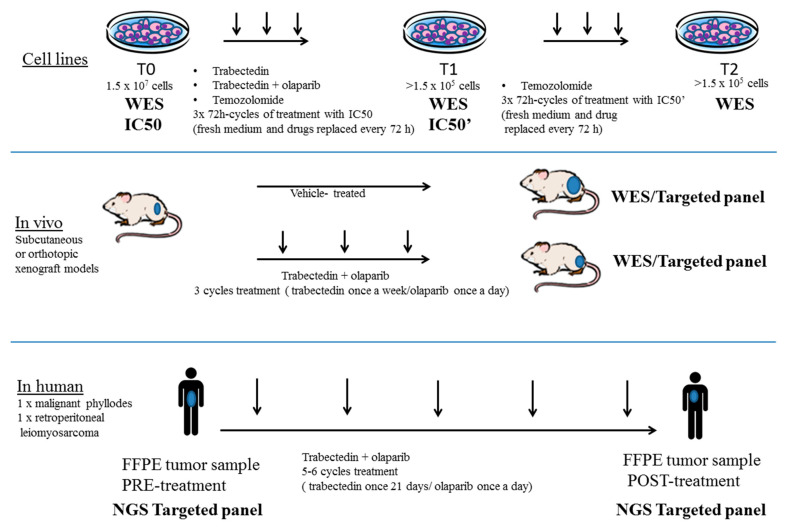
Experimental workflow on preclinical and translational platform. In vitro models (*n* = 9 for trabectedin + olaparib treatment): cell lines underwent 3 cycles of drug treatment as described in the Methods section followed by DNA extraction and WES analyses (T1). After 3 cycles of treatment (T1) no sufficient cells survived trabectedin and olaparib treatment. Temozolomide-treated cells (*n* = 5) underwent another cell viability test, showing increased resistance. After establishing a higher IC50 (IC50′), surviving cells (*n* = 1) underwent 3 novel cycles of temozolomide treatment, followed by DNA extraction (T2). In vivo models (*n* = 3): DMR, HT1080 and SJSA-1 were injected into the right flank or into the uterine wall (orthotopic model: DMR only) of NOD/SCID mice. After tumor engraftment, mice were treated with trabectedin + olaparib for 3 weeks or left untreated. Patient samples (*n* = 2): two patients enrolled in the TOMAS trial, affected by advanced sarcomas, were treated with trabectedin and olaparib combination at the recommended phase 2 dose. Extracted DNA (pre and post treatment) was analyzed by next-generation sequencing approaches: WES or targeted panel (Oncomine TML) scalable in low DNA concentration and FFPE conditions.

Human osteosarcoma (SJSA-1, CRL-2098, doubling time: 26 h), fibrosarcoma (HT-1080, CRL-121 doubling time: 22 h) and ovarian cancer (A2780, UWB1.289_BRCA1, OVCAR-8 and IGROV-1 doubling time: 22, 30, 25, 26 h, respectively) cell lines were obtained from the American Type Culture Collection (ATCC) and cultured following ATCC instructions. Human leiomyosarcoma cell line (DMR) was kindly provided by the group of Paola Spessotto (CRO-IRCCS, Aviano, Italy). One human liposarcoma cell line (402.91, doubling time: 21 h) was kindly provided by Dr. Pierre Aman (University of Gothenburg, Gothenburg, Sweden) and cultured in RPMI medium supplemented with 10% FBS. We obtained 402.91 shBRCA1 (doubling time 26 h) after infection with lentiviral particles, produced as described elsewhere [22]. Briefly, Dharmacon GIPZ™ Human BRCA1 shRNA Clone ID: V2LHS_280394 RHS4430-2001973 glycerol stock (Carlo Erba Reagents, Cornaredo, MI, Italy) was amplified in LB broth and selected by Ampicillin in LB-agar. The picked colony was amplified in LB broth and plasmids were purified by Qiagen Plasmids Maxy Prep Kit (Qiagen, Hilden, Germany). Packaging vectors pMDLg/pRRE pRSV-REV and pMD2.VSVG were used for lentiviral preparation. Efficiency of transduction was confirmed by detecting the percentage of GFP-positive cells using Cyan ADP Flow cytometer (Beckman Coulter, Brea, CA, USA) and by Western blot analysis (primary antibody: anti human BRCA1 #9010 Cell Signaling Technologies, Leiden, The Netherlands). Trabectedin (PharmaMar, Madrid, Spain), temozolomide (T2577, Sigma Aldrich, Merck, Darmstadt, Germany) and olaparib (SelleckChem, Munich, Germany) were dissolved in DMSO and stored at −80 °C until use. Untreated cell lines (time 0: T0) were subdued until cell viability assays for determining the concentration inhibiting 50% of cell viability (IC50) and to DNA extraction for whole exome sequencing (WES) analysis. Fifteen million cells were plated into 3 dishes of 150 mm diameter and after 24 h were treated with trabectedin, trabectedin plus olaparib or temozolomide at established IC50 for 72 h or left untreated. The treatment was repeated three times and if sufficient viable cells were spared by the drug treatment (at least 1% viable cells = 1.5 × 10^5^ viable cells), DNA was extracted and prepared for WES (T1). The surviving fraction was subdued in another cell viability assay to verify the occurrence of drug resistance and to establish the novel IC50 (IC50’). Resistant cells were subdued to another 3 cycles of treatment with the novel IC50’ and the surviving fraction underwent DNA extraction and WES analysis (T2) (Figure 1).

### 2.2. Cell Viability Assay and IC50 Determination

To determine the concentration inhibiting 50% of cell viability (IC50) of temozolomide or a trabectedin and olaparib combination, viability assays were performed using a Cell Titer-Glo^®^ kit (Promega Corporation Madison, WI, USA) after 72 h of drug treatment. Briefly, 2000 cells were seeded on 96-well plates and incubated for 24 h in their complete medium and subsequently for 72 h with scalar doses of temozolomide (1–0.0625 mM) or trabectedin (1–0.0625 nM) and olaparib (10–0.625 µM) as single agents or as constant combination. Cell Titer-Glo^®^ Reagent was added to each well for 15 min at room temperature in the dark to allow luminescence signal production. Synergy HT luminometer (Biotek Instruments, Winooski, VT, USA) and Gen5 v1.09 software (Biotek Instruments) were used for signal acquisition. IC50 with its 95% confidence intervals (95% CI), and drug synergism, expressed as combination index (CI) calculated at IC50 with its estimated standard deviation were obtained by using CalcuSyn software (Biosoft, Cambridge, UK) based on isobologram analysis. A dose–effect curve was obtained by GraphPad Prism 7.0.2 software (La Jolla, CA, USA) and analyzed by two-way ANOVA with Bonferroni’s correction.

### 2.3. Western Blot

To obtain total protein extracts, cells were detached with trypsin-EDTA in PBS (EuroClone, Pero, Milan, Italy), washed with cold PBS, and re-suspended at a concentration of 10^7^ cells/mL in Lysis Buffer 6 (R&D Systems, Minneapolis, MN, USA). Cells were mechanically lysed on ice with a pipette and rocked gently for 20 min at 4 °C and then clarified to remove cell debris. Protein concentration was determined by using a bicinchoninic acid assay (BCA Protein Assay, Thermo Fisher Scientific, Life Technology Italia, Monza, Italy) and absorbance levels were measured using the BioPhotometer (Eppendorf, Hamburg, Germany). A total of 30 µg of protein samples were resolved by electrophoresis on 4–20% mini-PROTEAN^®^TGX™ PreCast gels (Bio-Rad Laboratories, Hercules, CA, USA), and then transferred to a nitrocellulose membrane via the Trans-Blot^®^ Turbo™ Transfer System (Bio-Rad Laboratories). Non-specific sites were blocked with 5% milk for 1 h at room temperature after which the membranes were incubated overnight at 4 °C with anti-human BRCA1 (1/1000 dilution in TBS/Tween 0.2%/BSA 0.5%; Cell Signaling Technology, Leiden, The Netherlands) or anti-human β-Actin (1/1000 dilution TBS/Tween 0.2%/BSA 0.5%; Cell Signaling Technology, Leiden, The Netherlands) antibodies. Specific signals were visualized with HRP-conjugated secondary antibodies (Jackson Immuno Research Laboratories, West Grove, PA, USA) and detected using the Bio-Rad Chemidoc™ Touch Imaging System (Bio-Rad Laboratories) following exposure to Clarity™ Western ECL substrate (Bio-Rad Laboratories). Band intensity was quantified by ImageLab v5.2.1 (Bio-Rad Laboratories) using β-actin as normalizers.

### 2.4. Sarcoma Xenograft Models in NOD/SCID Mice

One million 402.91, 402.91shBRCA1, DMR, HT1080 and SJSA-1 cells were injected subcutaneously into the right flank or orthotopically in the uterine wall (4 × 10^5^ DMR) of 5-week-old female NOD/SCID mice. After tumor engraftment, mice were treated with three cycles of 25 mg/kg/day olaparib (by intraperitoneal injection, 5 days/week) and 0.05 mg/kg trabectedin (intravenous administration once a week) or left untreated for 21 days (17 for SJSA) as previously described [22] accordingly to protocols approved by the Institutional Ethics Review Board (IRB) and by the Italian Ministry of Health (Aut. Min. 178/2015-PR). At the end of the experiments, tumor tissues were snap-frozen in liquid nitrogen for further DNA extraction.

### 2.5. TOMAS Trial Patients

Two patients affected by advanced sarcomas (one relapsing retroperitoneal grade 3 leiomyosarcoma and one malignant phyllodes tumor with multiple lung metastases), progressing after anthracycline-based treatment and enrolled in the phase 1b clinical trial TOMAS [23], were treated with trabectedin 1.1 mg/m^2^ every 3 weeks plus olaparib 150 mg twice daily. After 5 and 6 cycles, respectively, both patients underwent surgery, having achieved a partial response according to RECIST 1.1. Archival FFPE samples at baseline and after surgery were collected for molecular analyses according to the TOMAS protocol.

### 2.6. DNA Extraction

Total DNA was extracted from cultured cells (at least 1.5 × 10^5^ cells) and from frozen tissues using the Maxwell^®^ RSC Blood DNA Kit AS1400 (Promega), from FFPE archival tumor biopsy and surgical excision, after microdissection of the tumor area, using the Maxwell^®^ RSC DNA FFPE Kit AS1450 (Promega), and the RSC Maxwell Instrument (Promega) in accordance with the manufacturer’s instructions. DNA purity was checked by NanoDropTM (Thermo Fisher Scientific, Life Technology Italia, Monza, Italy). DNA concentration was determined by the Qubit dsDNA HS (high sensitivity) assay kit (Thermo Fisher Scientific, Life Technology Italia) and the Qubit^®^ 3.0 Fluorometer (Thermo Fisher Scientific, Life Technology Italia). DNA fragmentation was assessed by gel electrophoresis and a 2100 Bioanalyzer High Sensitivity DNA assay Kit (Agilent Technologies, Agilent Technologies, Inc., Santa Clara, CA, USA).

### 2.7. Genetic Analyses after Whole Exome Sequencing and NGS Targeted Approach

Library preparation for whole exome sequencing analysis was performed using a minimum of 300 ng of DNA from tissue samples and cell lines. The library preparation was performed by IntegraGen SA (Evry, France) using the Twist Bioscience^®^ (South San Francisco, California, USA) Human Core Exome (Consensus CDS) + IntegraGen content, for a genomic target of 37 Mb. All libraries were sequenced on a Novaseq 6000 sequencer (Illumina) and 100–150 bp paired-end reads were generated with an average sequencing depth of 135X depth per exome and a coverage >98% at >50X. Genetic discovery analysis was performed by an in-house NGS pipeline [25,26] constructed for WES analyses of paired cancer genomes in order to call somatic variations, indels and copy number alterations. The Xenome tool [27] was used to evaluate mouse-derived reads and to remove mouse sequences in xenograft samples. Human-assigned reads were then mapped to assemble hg19 of the human reference genome using a BWA-mem algorithm with standard parameters. PCR duplicates were removed using the RMDUP command of SAM tools package. Reads with more than 3 mismatches and bases with a Phred Score <30 were filtered out. All mutations detected by reads as having a strand bias or exclusively by mismatch in head/tail of the read were discarded. Only mutations with a minimum depth of 5X and at least 10% allelic frequency were considered. Reads supporting *MUC6* and *MUC2* gene variations were filtered out because DNA sequences of the human mucin genes have not been completely resolved due to the repetitive nature of their central exon coding for Proline, Threonine and Serine-rich sequences [28]. BAM files were then analyzed by comparing pre-treatment and post-treatment samples to identify mutations and indels in post-treatment matched samples. Mutations and indels common to both samples were classified as pre-treatment, while alterations present in post-treatment but not in pre-treatment were classified as acquired. After verifying the quality control of sequencing parameters for all samples, we confirmed that the samples analyzed belonged to the matched post-treatment samples by comparing their single nucleotide polymorphisms. The identity of all cell lines and xenografts were confirmed using the unique allelic profile (based on Single Nucleotide Polymorphism Identification, SNP_ID) using the dbSNP-version 147. Only alleles with a fractional abundance above 20% and depth above 10X were considered. For annotation of germinal alterations of 341 DDRR genes, we filtered out synonymous mutations and mutations with fractional abundance less than 10%. PolyPhen-2 was used to annotate the prediction effect of the mutation on the protein coded by the genes. TMB was calculated from WES data, taking into consideration somatic mutations after data normalization on the specific covered target region of each sample [29,30]. Our methodology was set up on biopsies of metastatic colon tissue, as previously described [26,29,30,31]. The ratio of median gene depth to the median depth of the whole exome was defined as Gene Copy Number (GCN). For each gene, its GCN in the pre- and post-treatment samples and the corresponding copy number variation (ratio between matched GCNs) were reported. A circular binary segmentation (CBS) algorithm using DNAcopy R module was performed to cluster the gene copy-number alterations [25]. Samples purified from HT1080 cells, DMR uterine orthotopic and HT1080 subcutaneous xenografts, and FFPE tumor samples from TOMAS trial patients underwent targeted panel sequencing with Oncomine™ Tumor Mutation Load Assay (Thermo Fisher Scientific, Inc., Waltham, MA, USA). This amplicon-based target gene panel (409 genes) was properly designed to evaluate the TMB even in fragmented and usually impracticable FFPE DNA samples. The NGS libraries were built starting from a total of 20 ng of DNA using the IonChef™ System (Thermo Fisher Scientific, Life Technology Italia, Monza, Italy). Briefly, eight samples per run were loaded in a preparation plate containing the IonCode™ barcodes. The automated instrument performed 16 cycles of PCR with 16 min of annealing and extension. Eight prepared libraries were diluted to 50 pM, pooled and loaded on the Ion 540™ chip by means of the IonChef™ System. Sequencing was achieved using the Ion GeneStudio™ S5 Plus System (Thermo Fisher Scientific, Life Technology Italia, Monza, Italy). The panel results were optimized for a total of 8 to 10 million reads with a 500X median coverage. The reads were aligned to assemble hg19 of the human reference genome by the Torrent Suite™ v5.8 (Thermo Fisher Scientific, Life Technology Italia, Monza, Italy), generating the BAM files, which were transferred on the Ion Reporter Server™ v5.10 (Thermo Fisher Scientific, Life Technology Italia, Monza, Italy) for the TMB calculation using the Oncomine™ Tumor Mutation Load v2.0 workflow (Thermo Fisher Scientific, Life Technology Italia, Monza, Italy). To standardize the analyses and to reduce the impact of sequencing artifacts derived from the formalin fixation, we set the allele frequency limit of detection at 10% for all the samples. The TMB was calculated as the number of single nucleotide variants, small indel non-synonymous mutations divided with at least 60X of alternative read depth by the active genome (regions under 250X were excluded). Germline variants were filtered automatically by cross-referencing with UCSC common SNPs, ExAC, 10,000 Genomes, and 5000Exomes databases.

### 2.8. Mutational Signature Analyses

The analysis of mutational signatures using the “fitting” method can identify the best combination of the known signatures explaining the observed mutational profile of cell lines and xenografts. Many of these signatures have been associated with a defined etiology, including mutational profiles derived from exposure to different treatments. Genetic alterations with fractional abundance higher than 10% were used for mutational signature analysis. The profile of each genetic signature was calculated using the six substitution subtypes: C > A, C > G, C > T, T > A, T > C, T > G. In detail, all substitutions were referred to the pyrimidine of the mutated Watson–Crick base pair. The flanking 5′ and 3′ bases of each mutation were characterized and reported in a mutational catalogue using sigprofilerMatrixGenerator [32], as initially reported in [33,34]. Using the information of single nucleotide variants in the matrix, a series of mutational profiles were extracted, and genetic signatures were calculated using the MuSiCa tool [35] and Mutational Patterns package [36]. As reported, the mutational catalogue of each sample (M) may be considered as the composite of all the mutagenic processes that were active in the cancer cells. Then, mutational signature fitting estimated the contribution (E) of each mutational process specifying the percentage of mutations that could be attributed to each genetic signature (S) for each cell line/xenograft (such as M ≈ S × E). The reference v 2.0 of the COSMIC signature database was used for fitting analysis. Cosine similarity was calculated by MuSiCa [35] and Mutational Patterns tools [36] and used to evaluate the similarity between the original mutational catalogue of profiles (identified by the mutations in the sample) and the reconstructed mutational profile based on the optimal linear combination of all COSMIC signatures (identified after fitting) [36].

## 3. Results

### 3.1. Generation of a Preclinical and Translational Platform of Bone and Soft Tissue Sarcoma Cell Lines, Xenografts and Tumor Samples from Patients Treated with Trabectedin and Olaparib

We generated a panel of bone and soft tissue sarcoma cell lines comprising one leiomyosarcoma (DMR), one liposarcoma (402.91), one osteosarcoma (SJSA-1) and one fibrosarcoma (HT-1080) treated with at least three cycles of trabectedin and olaparib combination or with temozolomide as a control (as described in the Methods section), obtaining a cell surviving fraction (>1%) sufficient for DNA extraction and WES analysis (>1.5 × 10^5^ viable cells, Figure 1).

**Figure 2:**
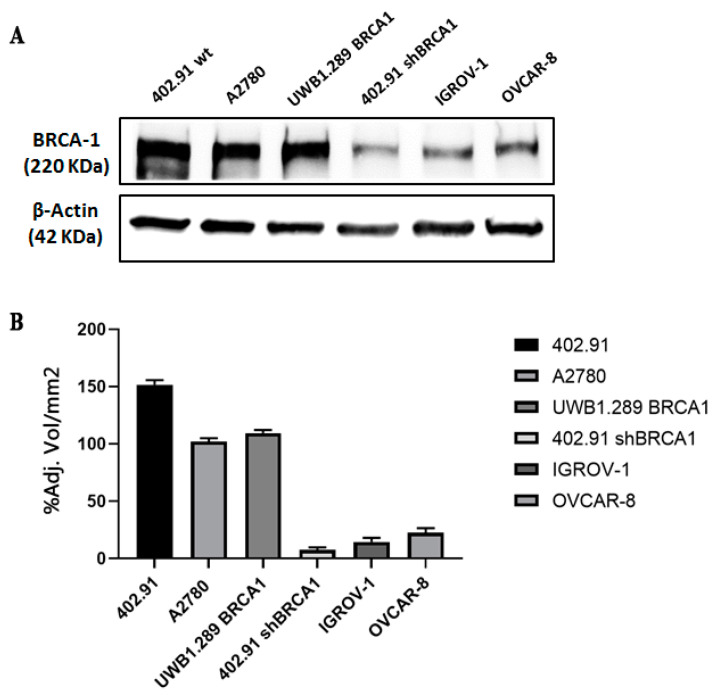
BRCA1 protein expression in cell lines. (**A**) Representative Western blot analysis of BRCA1 expression in BRCA1 proficient (402.91 wild type (wt), A2780, UWB1.289 BRCA1) and BRCA1 defective cells (BRCA1 stable silenced 402.91 (402.91 shBRCA1), IGROV-1 and OVCAR-8). β-actin was used as loading controls. (**B**) Densitometric analysis of protein band intensity normalized on housekeeping controls expressed as mean value of three replicates ±standard deviations of the percentage of adjusted volume/mm^2^.

In order to include a BRCA1 defective control, we generated a stable, silenced BRCA1 402.91 liposarcoma cell line (402.91 shBRCA1) and we completed the panel with BRCA1 defective (IGROV-1 and OVCAR-8) and BRCA1 proficient (A2780 and UWB 1.289_BRCA1) ovarian cancer cell lines. The expression of BRCA1 protein in the aforementioned cell lines was shown in Figure 2 and in Figure S1.

In Table 1, we summarized the drug concentration inhibiting 50% of cell viability for each cell line established after 72 h of treatment (72 h IC50). In particular, we observed that 402.91 and DMR cells were the most sensitive to trabectedin and olaparib combination followed by SJSA-1, while HT-1080 cells were the most resistant ones.

We observed that the sensitivity to trabectedin and olaparib as single agents was higher for 402.91shBRCA1 cells when compared to their parental counterpart (402.91; Table 1). Of note, in 402.91shBRCA1 cells, these two drugs did not have a synergistic effect (combination index >1), possibly due to the high activity of both single agents. As shown in Table 1, IC50 values of trabectedin and olaparib combination varied among different ovarian cell lines. Each cell line was treated with the established 72 h IC50 of the combination. Surviving fractions were collected at the end of the third cycle of treatment and underwent DNA extraction and WES (T1). No cell lines but SJSA-1 gave rise to sufficient surviving fraction (>1.5 × 10^5^ viable cells), to be challenged with further cycles of treatment with trabectedin and olaparib. We challenged all sarcoma cell lines with temozolomide and, once again, SJSA-1 was the least sensitive to temozolomide (Table 2). In particular, SJSA-1 cells’ treatment with the established temozolomide 72 h IC50 (677 µM) gave sufficient surviving fraction (>1.5 × 10^5^ viable cells) after the third cycle of treatment (T1). This fraction (now referred as SJSA-1 T1 cells) underwent further cell viability assays, showing increased drug resistance. Namely, the 72 h IC50 was 677 µM with a 95% confidence interval ranging from 486 to 943 µM in wild-type SJSA-1 and 1499 µM (IC50’) with a 95% confidence interval ranging from 1051 to 2137 µM in SJSA-1 T1 cells (Table 2).

The SJSA-1 T1 cells were further treated with 3 × 72 h cycles of treatment, with temozolomide at IC50’ (1499 μM) reaching the second time point of DNA extraction (T2).

The translational platform was further enriched with in vivo mouse models, derived from the human sarcoma cell lines tested in vitro. In particular, 402.91, 402.91shBRCA1, DMR, SJSA-1 and HT1080 cell lines were injected subcutaneously into the right flank of NOD/SCID mice. DMR cells were also implanted orthotopically into the uterine wall of an additional cohort of female NOD/SCID mice. All cell lines apart from 402.91 and 402.91shBRCA1 were successfully engrafted in NOD/SCID mice, and animals were treated with three cycles of trabectedin and olaparib or left untreated.

**Figure 3:**
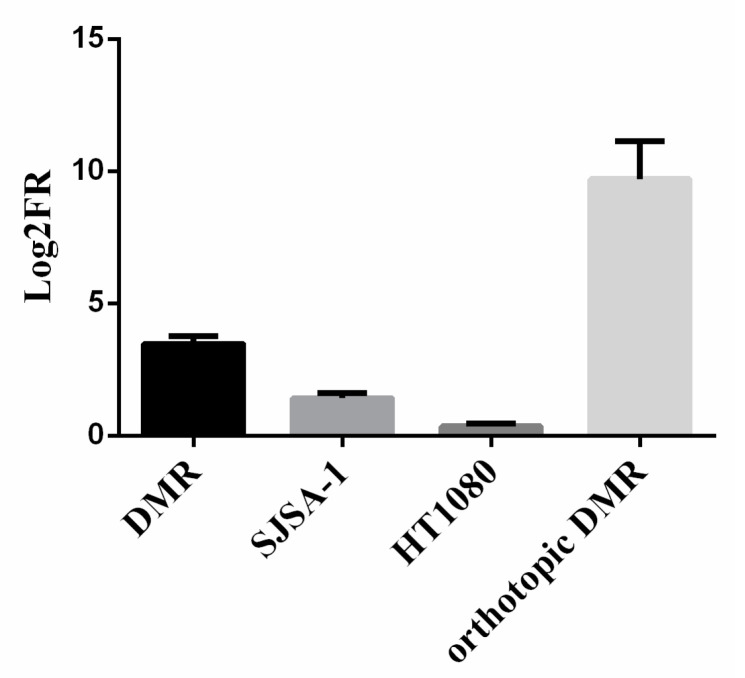
Antitumor effect in DMR, SJSA-1 and HT1080 xenograft models and DMR orthotopic model treated with trabectedin and olaparib combination compared to untreated controls. Five mice per group (control and combination treated) were included in the experiment. Log2FR = Log2 fold-reduction in tumor volume as compared to untreated controls. Results are depicted as mean of Log2FR ± standard deviation.

In the three successfully engrafted subcutaneous models (DMR, SJSA-1 and HT1080), the trabectedin and olaparib combination reduced tumor volume by 13.3, 2.8, and 1.4-fold compared to untreated controls, respectively (Figure 3). In the DMR orthotopic model, the tumor growth was revealed by in vivo imaging techniques and the reduction induced by the combination was 2550-fold of the photon intensity compared to untreated controls (Figure 3). No sign of toxicity was observed in terms of weight reduction and after gross necropsy.

To complete our preclinical and translational platform with tumor samples derived from patients treated with trabectedin and olaparib combination, we obtained FFPE tumor tissue before and after treatment. One metastatic malignant phyllode and one retroperitoneal leiomyosarcoma were treated with five and six cycles of trabectedin and olaparib combination, achieving partial responses and making them eligible to complete surgery. All samples before and after treatment were analyzed by NGS to investigate both mutational status of DDRR genes and TMB (Figure 1).

### 3.2. Genetic Analysis of DNA Damage Response and Repair (DDRR) Genes and Copy Number Alteration in a Preclinical and Translational Platform of Bone and Soft Tissue Sarcomas Treated with Trabectedin and Olaparib

We explored DDRR pathways by analyzing mutations and CNV after WES or targeted panel NGS in our preclinical and translational platform of bone and soft tissue sarcomas before and after the treatment with trabectedin and olaparib combination. After quality controls of all WES outputs, single nucleotide polymorphisms (as annotated in dbSNPs v.147) were analyzed comparing treated and untreated samples, confirming concordance (percentage higher than 95% of concordance, Supplementary Table S1).

Based on Gene Ontology, we selected 341 DDRR genes (listed in Supplementary Table S2) for their role in the base excision repair (BER), DNA damage response (DDRR), Fanconi anemia homologous recombination (HRFA), nucleotide excision repair (NER), non-homologous end joining (NHEJ) and DNA mismatch repair (MMR) pathways and we investigated their mutational status. Moreover, we predicted the possible functional impact of identified amino acid substitutions on stability and function using the Polyphen-2 tool excluding common SNPs. In SJSA-1 cells, we observed three “probably damaging” nonsynonymous amino acid substitutions in BER genes (*MUTYH*, *NEIL1* and *NEIL2*), five in DDRR genes (*SENP7*, *INO80D*, *UBA7*; *UBR5*, *Y1AP1*), eight in HRFA genes (*CNTROB*, *DDX11*, *FANCD2*, *MCMDC2*, *NSMCE2*), one in the MMR gene (*RFC1*), two in NER genes (*XPC*, *BIVM-ERCC5*). In DMR cells, two genes belonging to DDR (*UPF1, TRRAP*) displayed mutations and one gene (*DCAF4L2*), belonging to the NER pathway. In wild-type and shBRCA1 402.91 cells, we observed four mutations in DDR genes (*CINP*, *HERC2*, *HUS1B*, *SENP7*), five mutations in four HRFA genes (*DDX11*, *NBN*, *PARP4*, *SLX4*), one in the MMR gene (*MSH4*) and two in NER genes (DCAF4, MC1R). In one patient tumor sample (TOMAS B), mutations were found in both DDRR (*TP53*) and HRFA (*WRN*) pathways. No relevant modulation in the allelic frequency of the identified mutations were induced by trabectedin and olaparib treatment both in cell lines and in xenograft models, while a negligible increase in *WRN* mutations was observed in the sample derived from patient TOMAS B (Table 3).

**Figure 4:**
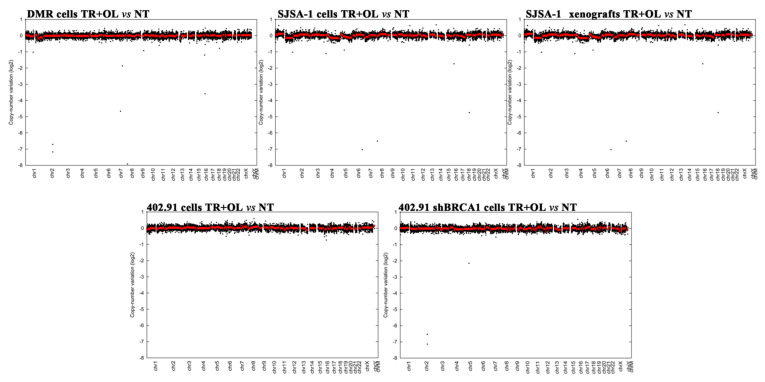
Copy number alterations calculated on WES data output after trabectedin + olaparib (TR + OL) treatment in DMR, SJSA-1, and SJSA-1 xenografts, 402.91, and 402.91 with stable silenced BRCA1 compared to relative untreated controls. Results are shown as Log2 variations in copy number (treated vs. untreated samples), plotted against chromosomal locations.

To further verify the absence of genomic alterations after trabectedin and olaparib treatment, we performed copy number alteration analysis in sarcoma samples, comparing samples before and after treatment. No significant gene amplifications or copy loss, consistent with the loss of heterozygosity were found after treatment with trabectedin and olaparib in both cell lines and xenografts models compared to the parental counterpart (Figure 4).

### 3.3. Treatment with Trabectedin and Olaparib Combination Did Not Influence Single-Base Substitution (SBS) Signatures

**Figure 5:**
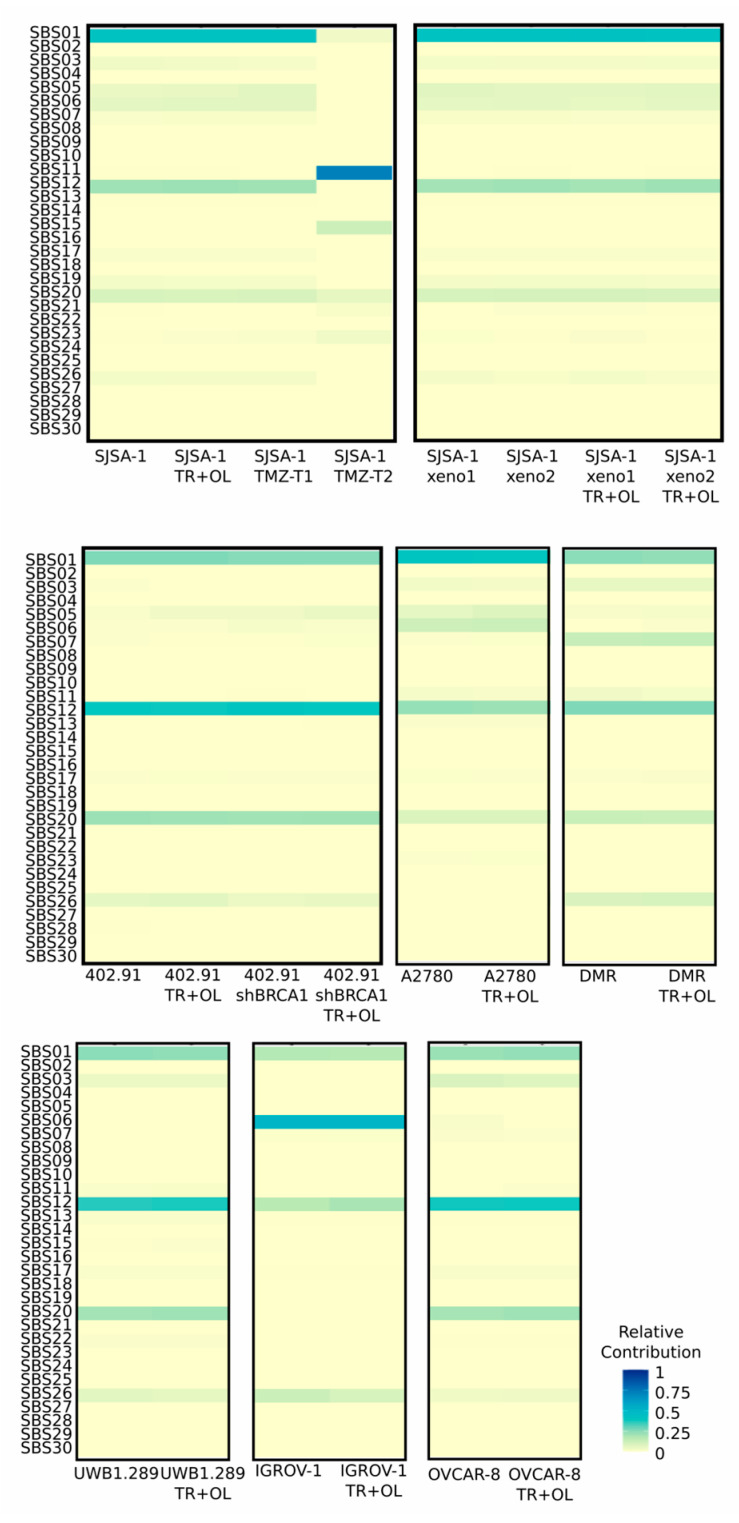
Mutational signatures analysis. Heatmap of normalized signature performed using COSMIC v2.0 as reference for the fitting analysis. Results are shown as the relative contribution of the specific SBS signature in treated and untreated samples for each specific cell line and treatment type. TR + OL: after three cycles of treatment with trabectedin + olaparib combination. TMZ: after temozolomide treatment.

To mechanistically determine the impact of the PARP1 inhibitor olaparib and trabectedin combination on samples/cell lines, we performed mutational signature analysis, investigating each cell line and xenograft before and after olaparib and trabectedin combination. We also analyzed cell lines treated with the drug temozolomide as a positive control, because the signature SBS11 (in the COSMIC mutational signature reference) emerges after temozolomide treatment [33,34]. As shown in Figure 5, trabectedin and olaparib combination did not relevantly impact the mutational profiles of each cell line and xenograft treated with the olaparib and trabectedin combination. Conversely, as expected, temozolomide treatment induced a strong increment in Signature SBS11 in SJSA-1 T2 cells, showing a normalized score of 0.72. Namely, this score is related to the increasing number of mutations and specifically, it showed that 72% of the mutations detected were predicted as induced by temozolomide.

### 3.4. Trabectedin and Olaparib Treatment Did Not Impinge on Tumor Mutational Burden

**Figure 6:**
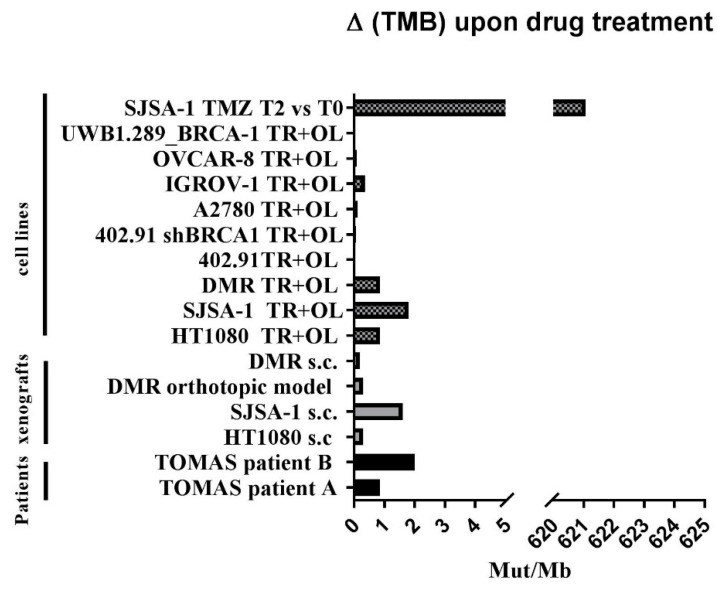
Variations in tumor mutational burden (ΔTMB) in a preclinical and translational platform of bone and soft tissue sarcomas treated with trabectedin + olaparib (TR + OL). TMB was calculated on somatic mutations identified on WES or targeted panel sequencing (Oncomine TML). Results are shown as alterations in the number of mutations per megabase, in treated samples vs. their untreated/baseline controls.

ΔTMB was reported in cell lines and xenografts analyzed by WES or by the targeted Oncomine TML™ panel evaluating the somatic variations acquired after drug treatment. A very low ΔTMB was found after trabectedin and olaparib both in BRCA1/2-proficient and -defective cell lines, and in sarcoma xenografts, ruling out a mutagenic effect of the drug combination (Figure 6). Consistently, TMB analysis in tumor samples derived from patients treated with trabectedin and olaparib (five and six cycles, respectively), confirmed the absence of ΔTMB. As expected, SJSA-1 T2 cells (resistant to temozolomide) showed a massive increase in TMB, compared to baseline SJSA-1 T0 cells (Figure 6), confirming the result obtained by mutational signature analysis.

**Figure 7:**
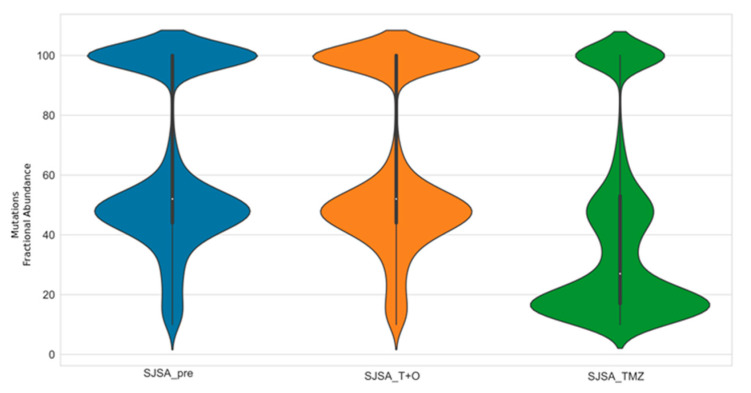
Clonality at baseline, after trabectedin + olaparib combination and after temozolomide treatment in the SJSA-1 cell line. The distribution of mutations fractional abundance for each treatment setting was showed SJSA_pre = SJSA-1 before treatment; SJSA_T + O = SJSA-1 after trabectedin + olaparib combination; SJSA_TMZ = SJSA-1 after temozolomide treatment.

Notably, no clonality variation after trabectedin + olaparib combination was found, while in SJSA-1 cells treated with TMZ, it was possible to detect the appearance of a new clonal population due to TMZ treatment in SJSA-1 cells treated with TMZ (Figure 7).

## 4. Discussion

We explored the potential impact of an innovative therapeutic combination with trabectedin and olaparib, on the variation in TMB in advanced bone and soft tissue sarcomas, usually considered as “immune-cold tumors”.

Indeed, compared to several epithelial tumors, bone and soft tissue sarcomas are not usually caused by mutagenic carcinogens known to introduce a high number of mutations in the human genome (such as tobacco smoke in lung cancer or UV light in melanoma) [37]. Furthermore, several mesenchymal tumors carry driver translocations (for instance, FUS-CHOP t(12; 16)(q13; p11) in myxoid liposarcoma [38] or EWS/FLI1 t(11; 22)(q24; q12) and the likes in Ewing’s sarcoma [39]) and are characterized by a relatively simple karyotype. A noteworthy exception is represented by gastrointestinal stromal tumors, wherein activating mutations in either KIT or PDGFRα genes drives tumor proliferation. In this context, trying to boost TMB by therapeutics in bone and soft tissue sarcomas could be a promising avenue to unleash response to immunotherapeutic strategies.

It should be emphasized that TMB is not a flawless predictive biomarker for immunotherapy response, but it is a useful proxy in terms of reproducibility, timing and large-scale feasibility of its evaluation [37]. In fact, not all nonsynonymous mutations will result in neoantigen generation, and not all novel peptides are effective immunogenic targets, adequately presented to the immune system [40].

By means of a robust preclinical platform, along with tumor samples of patients treated with this combination, we assessed both the mutational status of the DNA repair machinery and TMB before and after treatment with trabectedin and olaparib. We observed no change in mutational profiles, DDRR defects, copy number alterations and no relevant increase in TMB in either the preclinical or clinical setting. Overall, these data suggest that the combination of trabectedin and olaparib does not increase TMB, after three (preclinical) or five/six (patients) cycles of drug treatment. This information is reassuring regarding the feared risk of myelodysplasia/leukemia [19] reported with PARP1 inhibitors, but, at the same time, indicates that tumor-specific neoantigens are unlikely to be generated with this combination. Therefore, any future integration with ICI should be based on microenvironment modifications, rather than on TMB increase. This somewhat unexpected finding brings novel information to the foreground: chemotherapy and target therapy combination may end up in different genetic consequences, according to proficient or deficient pathways characterizing each specific tumor cell. Immune therapy with ICI represents a breakthrough in today’s cancer treatment. However, in the sarcoma setting, only a minority of patients can really benefit from this intervention, at least in terms of overall survival and progression-free survival [24,41,42,43,44]. Thus, an extraordinary effort was made to both identify predictive key features in responsive tumors and modify the tumor/tumor microenvironment, aiming at improving patients’ selection and enlarge the number of patients rationally eligible for ICI. Initially, PDL-1 expression was the first paradigmatic marker in candidate patients of immunotherapy. Later on, other biomarkers of response emerged, such as TMB, immune infiltrates and DDRR defects that showed a direct correlation with outcome after treatment with ICI [1,5,6,7,37,45]. These findings explained why sarcomas seldom respond to ICI and prompted studies to dissect sarcoma heterogeneity and attempts to modify the tumor and its microenvironment, making them more suitable for ICI. In this perspective, the combination of trabectedin and olaparib had a theoretically strong rationale: trabectedin and its derivatives modify the microenvironment, angiogenesis, and macrophage subsets [46,47]. The inhibition of PARP1, a key actor of DDRR, perpetuates this damage by impeding DNA repair [22]. In fact, we recently demonstrated that PARP1 inhibition enhances trabectedin-induced DNA damage and that treatment with this combination is feasible and active in advanced sarcoma patients [22,23]. In the present work, we explored whether tumor cells spared by this treatment might accumulate DNA damage and potentially be primed for subsequent immunotherapy with ICI. We assessed TMB, DDRR defects, gene copy number and mutational signatures in a preclinical and translational platform of bone and soft tissue sarcomas, treated with trabectedin and olaparib. At the end of these experiments, tumor cells spared by the combination both in preclinical and in patients’ tumor samples did not show any modification in DDRR gene status, gene copy number or increase in TMB, nor did they relevantly impact on the mutational profiles of each cell line and xenograft treated with the olaparib and trabectedin combination.

Given the fact that TMB measure is relative and that the number of clonal mutations is also relevant [48], we evaluated the clonality of our samples before and after treatment. We found no clonality variation in all samples treated with trabectedin + olaparib combination, while in our positive control (temozolomide treatment), the appearance of a new clonal population was observed. This is in line with our findings, highlighting the robustness of the selected control. Indeed, the increase in TMB could be linked to the intratumor heterogeneity increase (ITH). ITH is a known foe of molecular therapies because targeting a subpopulation of cancer cells (with a specific mutation) will lead to the Darwinian selective emergence of resistant clones [49]. In the immunotherapy field, this point is also under discussion. ITH appears as more a friend of immunotherapy than a foe, because it can be associated with the appearance of novel tumor antigens. In lung cancer [50] and melanoma [51], the data seem to be contradictory [52]: the high number of cancer neoantigens—created by strong subclonal expansions—attenuates anti-tumor immune responses to checkpoint blockade therapy. Recent data support these findings, suggesting that it is the percentage of tumor cells bearing each antigen within a tumor and not the absolute number of antigens that determines the efficacy of immune response [53]. In our study, we observed no differences either in clonal population distribution or in terms of ITH increase, after trabectedin + olaparib combination, differently from what we observed in the positive control (temozolomide treatment), in which novel clonal population emerged.

One intriguing aspect of our work was the observation that BRCA1 silencing in 402.91 cells (402.91shBRCA1) resulted in higher sensitivity to single agents, and consequent loss of evident synergistic effect. Indeed, it would be interesting to study the consequences of abrogated BRCA1 expression in in vivo models, treated with trabectedin + olaparib combination. This might reveal tumor microenvironment contributions to drug response and acquired genetic alterations in the presence or absence of BRCA1 loss.

In summary, on one hand, we confirmed that this treatment was safe and no hypermutation was identified in both studied models and patients. This finding is reassuring regarding PARP1i-related concerns about an increased risk of myelodysplasia/leukemia [19]. On the other hand, at least in terms of TMB, this combination does not seem an ideal induction therapy to ICI strategy. We acknowledge that other important aspects of crosstalk with the immune system might be evoked by this combination, such as modulation of the tumor microenvironment by both trabectedin and PARP1 inhibition [11,46,47] and activation of the stimulator of interferon genes (STING) pathway [54].

Other strategies to boost ICI response could also include multimodality treatment with chemotherapy and/or radiation therapy: both can increase TMB and, at the same time, improve local disease control. Furthermore, radiotherapy induces radiation-elicited T-cell activation (“radiation-induced viral mimicry”, through STING pathway activation), and enhances cancer cell immunogenicity [55]. Genotoxic chemotherapy can generate similar effects, but not all chemotherapeutic agents have equal potency. For instance, the effect has been demonstrated with anthracyclines and platinum-derived chemotherapy [56]. Radiation therapy has been studied separately both in association with trabectedin in sarcomas, at the preclinical [57] and clinical [58] level, and with PARP1-inhibitors, which are known radiosensitizers [59]. Toxicity issues should always be considered in multimodality treatment, and moreover, the radiation dose needed to induce a significant TMB increase is still a matter of debate [60]. Indeed, conventional radiotherapy (e.g., 60 Gy in 2 Gy-fractions) can introduce approximately one mutation per Megabase in the tumor genome, whilst techniques using larger doses per fraction (as stereotactic body radiotherapy) may obtain more relevant results [60].

We acknowledge that our work has limitations—in particular, the low number of available samples. Concerning the preclinical platform, it should be noted that establishing sarcoma PDXs is often challenging, also for the rarity of the disease. Regarding clinical, patient-derived samples, the only way to assess TMB variation was to collect samples from patients with very good responses, i.e., candidates for surgery after treatment. Otherwise, no post-treatment sample would have been available as the clinical study protocol did not envision post-progression biopsies. Only two samples were obtained, but given the fact that advanced/metastatic and pretreated sarcomas were enrolled, achieving two significant partial responses should already be considered as an exceptional occurrence. Exploring these findings in a larger patient cohort would be of interest to further confirm our results.

Finally, immuno-proficient preclinical models and a translational study on a larger cohort of treated patients will be instrumental for further investigations on these potentially actionable aspects. Moreover, a small subset of DDRR-defective tumors might be even more prone to both trabectedin + olaparib and ICI treatment, and molecular characterization of tumor samples could be a powerful tool for patient selection.

## 5. Conclusions

In conclusion, trabectedin and PARP1i treatment do not increase the TMB of sarcomas. Of course, it must be emphasized that sarcomas have more than eighty different histotypes and our conclusions may not apply to other rare histotypes. Other kinds of alkylating drugs, such as temozolomide, have been implicated in hypermutation, but the less safe toxicity profile may impinge on the clinical applicability of this drug, especially in combination with PARP1i [61]. Evaluating both genetic profiles and microenvironment changes in immunologically “cold” tumors, such as sarcomas, after conventional chemotherapy could be instrumental for developing novel insights to improve concomitant or sequence combination treatment with ICI.

## Figures and Tables

**Table 1:** Concentration inhibiting 50% of cell viability (IC50) and 95% confidence intervals (95%CI).

Cell Line	IC50 Trabectedin (95% CI) nM		IC50 Olaparib (95% CI) µM		Combination Combination Index ± Est. SD
	as SINGLE Agent	in Combination with Olaparib	as Single Agent	in Combination with Trabectedin	
DMR	0.57 (0.45–0.64)	0.13 (0.12–0.14)	9.02 (8.11–10.23)	1.30 (1.18–1.43)	0.47 ± 0.06
SJSA-1	0.25 (0.16–0.39)	0.18 (0.15–0.19)	14.55 (6.66–29.64)	1.80 (1.52–1.93)	0.86 ± 0.11
HT-1080	0.75 (0.68–0.81)	0.60 (0.59–0.64)	>20	6.04 (5.92–6.42)	0.89 ± 0.15
402.91	0.46 (0.37–0.54)	0.12 (0.11–0.14)	7.12 (5.53–7.91)	1.84 (1.37–2.68)	0.50 ± 0.12
402.91 shBRCA1	0.19 (0.12–0.28)	0.14 (0.04–0.53)	2.92 (2.19–3.89)	1.40 (0.38–5.27)	1.24 ± 0.22
A2780	0.41 (0.29–0.58)	0.20 (0.12–0.32)	8.73 (7.22–10.55)	1.97 (1.22–3.18)	0.7 ± 0.11
UWB1.289 BRCA1	0.44 (0.30–0.66)	0.41 (0.37–0.47)	10.36 (5.37–19.98)	4.18 (3.67–4.75)	1.35 ± 0.17
OVCAR-8	0.38 (0.12–1.20)	0.16 (0.04–0.70)	9.77 (6.06–15.74)	1.62 (0.37–7.04)	0.60 ± 0.18
IGROV-1	0.62 (0.39–0.99)	0.326 (0.18–0.58)	21.04 (13.59–32.57)	3.26 (1.82–5.85)	0.68 ± 0.14

Combination index ± estimated standard deviations (Est. SD) calculated at IC50 for trabectedin and olaparib combination in cell lines of different histotypes: leiomyosarcoma (DMR), osteosarcoma (SJSA-1), fibrosarcoma (HT-1080), liposarcoma (wild-type 402.91 and shBRCA1 402.91) and ovarian cancer (A2780, UWB1.289 BRCA1, OVCAR-8, IGROV-1). IC50 was calculated for each cell line after 72 h of treatment with serial dilutions of trabectedin (1–0.125 nM) and olaparib (10–1.25 µM) as single agents or in combination (constant ratio 1:10,000). Each experiment consisted of eight technical replicates and was repeated three times.

**Table 2:** IC50 and 95% CI after 72 h-temozolomide treatment.

Cell Line	IC50 Temozolomide (95% CI) μM
DMR	594 (499–706)
HT-1080	489 (416–575)
402.91	151 (133–172)
402.91 shBRCA1	242 (208–281)
SJSA-1	677 (486–943)
SJSA-1 T1	1499 (1051–2137)

Concentration inhibiting 50% of cell viability (IC50) after 72 h of treatment with serial dilutions of temozolomide (2000–125 μM) as single agents in sarcoma cell lines of different histotypes: leiomyosarcoma (DMR), osteosarcoma (SJSA-1), fibrosarcoma (HT-1080), liposarcoma (wild-type 402.91 and shBRCA1 402.91. SJSA-1 T1 cells were generated from SJSA-1 after three cycles of 72 h treatment with 677 µM of temozolomide. Each experiment consisted of eight technical replicates and was repeated three times.

**Table 3:** DDRR genes in sarcoma cells and tumor samples from patients affected by nonsynonymous “probably damaging” mutations as predicted by the PolyPhen-2 tool. WES and targeted panel sequencing results were analyzed searching for single nucleotide non-synonymous substitutions not previously described as SNP.

**Pathway**	**Gene**	**Base Change**	**Amino Acid Change**	**Mean Allelic Frequency**				
				**SJSA-1 Untreated Cells**	**SJSA-1 TR + OL Treated Cells**	**SJSA-1 Untreated Xenografts**		**SJSA-1 TR + OL Treated Xenografts**
BER	*MUTYH*	c.T538C	p.Y180H	48.76	50.00	39.67		39.58
	*NEIL1*	c.C826A	p.P276T	48.28	36.73	52.94		40.60
	*NEIL2*	c.C307T	p.R103W	65.67	63.89	66.47		67.76
DDR	*SENP7*	c.G1909T	p.D637Y	48.60	49.21	48.34		46.57
	*INO80D*	c.C2876G	p.S959C	49.05	44.97	45.58		44.19
	*UBA7*	c.A2450G	p.H817R	44.05	50.00	48.55		57.70
	*UBR5*	c.G3850A	p.A1284T	46.02	43.45	49.16		53.05
	*YY1AP1*	c.C385T	p.L129F	40.85	42.10	48.72		41.65
HRFA	*CNTROB*	c.G605T	p.R202L	45.34	55.05	44.74		44.67
	*CNTROB*	c.G1316A	p.R439Q	43.91	51.23	48.23		44.21
	*DDX11*	c.C1040A	p.A347D	37.70	42.21	38.87		34.82
	*FANCD2*	c.T1367G	p.L456R	26.66	25.67	25.12		24.77
	*FANCD2*	c.A1868C	p.Q623P	32.46	29.70	36.02		31.80
	*MCMDC2*	c.C1570T	p.P524S	28.06	29.59	29.16		31.35
	*NSMCE2*	c.T377G	p.F126C	45.10	47.71	52.83		45.60
	*SLX4*	c.A1637G	p.Y546C	53.01	51.25	47.00		52.70
	*SLX4IP*	c.G779A	p.G260E	46.67	38.86	41.86		48.35
MMR	*RFC1*	c.G2076C	p.E692D	100.00	98.00	100.00		98.72
NER	*XPC*	c.T860G	p.F287C	53.38	46.67	50.49		49.13
	*BIVM-ERCC5*	c.G2003A	p.R668H	17.62	15.28	13.29		13.71
**Pathway**	**Gene**	**Base Change**	**Amino Acid Change**	**Mean Allelic Frequency**				
				**DMR Untreated Cells**	**DMR TR + OL Treated Cells**			
DDR	*UPF1*	c.G2204A	p.G735D	100.00	98.56			
	*TRRAP*	c.C2165T	p.S722F	17.62	15.85			
NER	*DCAF4L2*	c.G250T	p.D84Y	53.38	47.24			
**Pathway**	**Gene**	**Base Change**	**Amino Acid Change**	**Mean Allelic Frequency**				
				**TOMAS Untreated Patient B**	**TOMAS TR + OL Treated Patient B**			
DDR	*TP53*	c.G248A	p.S215N	42.44	47.76			
HRFA	*WRN*	c.G250T	p.D84Y	<10%	15.38			
**Pathway**		**Gene**	**Base Change**	**Amino Acid Change**	**Mean Allelic Frequency**			
					**402.91** **Untreated Cells**	**402.91** **TR + OL Treated Cells**	**402.91 shBRCA1** **Untreated Cells**	**402.91 shBRCA1** **TR + OL Treated Cells**
DDR		*CINP*	c.C159G	p.N53K	49.26	52.54	46.71	51.45
		*HERC2*	c.A32G	p.Q11R	0	27.78	11.76	16.67
		*HUS1B*	c.C390A	p.H130Q	51.36	45.11	55.67	54.41
		*SENP7*	c.A1836T	p.Q612H	53.73	54.55	56.67	52.54
HRFA		*DDX11*	c.G1819C	p.A607P	9.38	5.66	14.15	10.16
		*DDX11*	c.C556T	p.R186W	16.39	19.12	16.05	13.68
		*NBN*	c.A511G	p.I171V	50.43	56.96	48.81	46
		*PARP4*	c.G2695A	p.A899T	48.89	33.66	39.71	48.91
		*SLX4*	c.C3812T	p.S1271F	49.80	45.28	52.89	51.01
MMR		*MSH4*	c.A1766G	p.Y589C	52.75	53.42	39.33	35.19
NER		*DCAF4*	c.C1034G	p.S345C	45.88	45.38	40.56	41.95
		*MC1R*	c.C478T	p.r160W	99.22	85.29	58.24	59.83
			c.G178T	p.V60L	0	9.80	40.56	38.36

## Data Availability

Data are available upon reasonable request.

## Supplementary Materials

The following are available online at https://www.mdpi.com/article/10.3390/cancers13246295/s1. Table S1: Results of single nucleotide polymorfism identification (SNP_ID) for samples. Table S2: list of DDRR genes analyzed on WES data. Figure S1: Uncropped Western Blot Images.
