# Supplementary figures and images for: PARP1 Inhibitor and Trabectedin Combination Does Not Increase Tumor Mutational Burden in Advanced Sarcomas—A Preclinical and Translational Study
Source: Cancers (Basel). 2021 Dec 15;13(24):6295. doi: 10.3390/cancers13246295 (PMC8699802; doi:10.3390/cancers13246295)

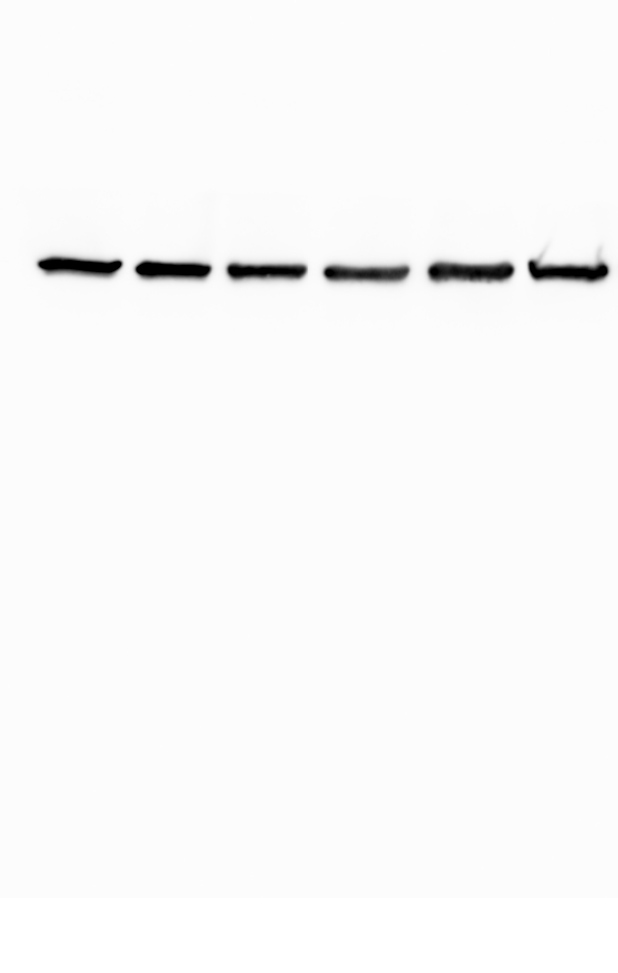

Supplement: Supplementary file 1 [file cancers-13-06295-s001.zip › actin full image.jpg]

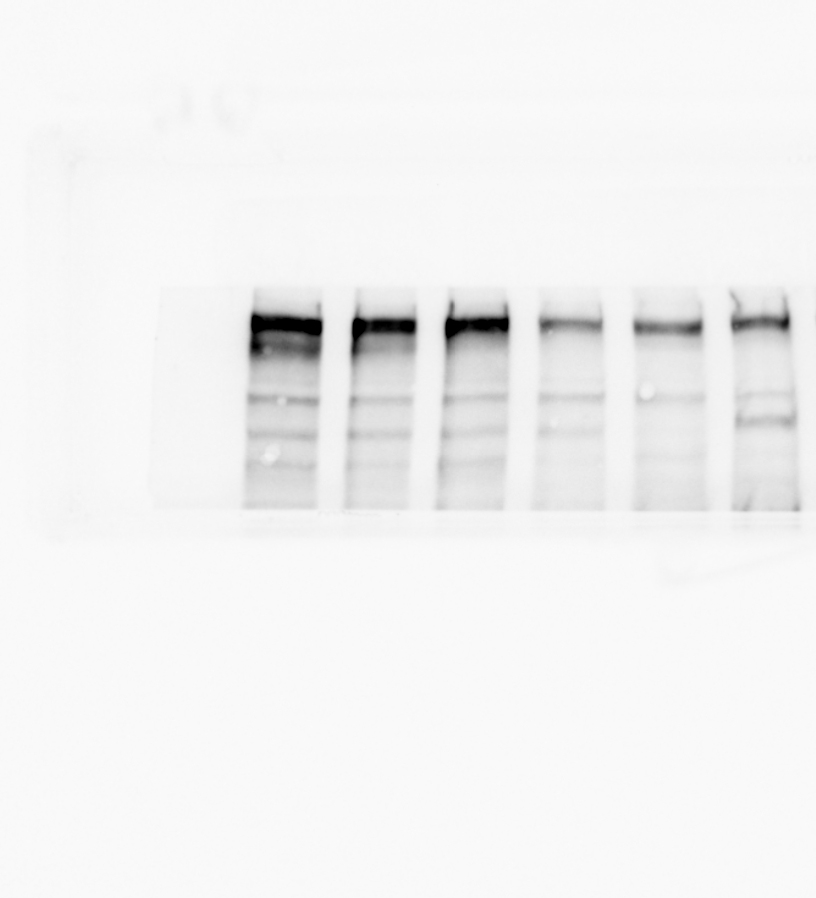

Supplement: Supplementary file 1 [file cancers-13-06295-s001.zip › brca1 full image.jpg]

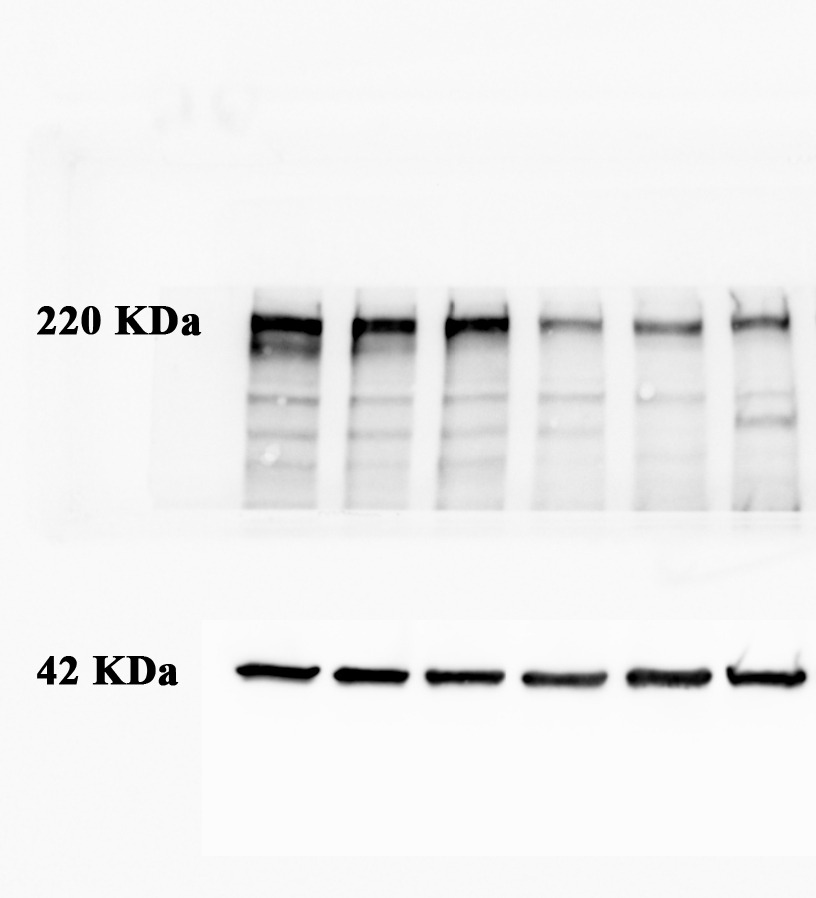

Supplement: Supplementary file 1 [file cancers-13-06295-s001.zip › Figure S1.tif]
